# An electromyographic analysis of shoulder muscle activation during push-up variations on stable and labile surfaces

**DOI:** 10.4103/0973-6042.40456

**Published:** 2008

**Authors:** Jaspal S. Sandhu, Shruti Mahajan, Shweta Shenoy

**Affiliations:** Department of Sports Medicine and Physiotherapy, Guru Nanak Dev University, Amritsar, India

**Keywords:** Electromyography, labile, rehabilitation, stable, strength, swiss ball

## Abstract

**Background:**

Numerous exercises are used to strengthen muscles around the shoulder joint including the push-up and the push-up plus. An important consideration is the addition of surface instability in the form of swiss ball for rehabilitation and strength. The justification for the use of the swiss ball is based on its potential for increasing muscular demand required to maintain postural stability and for improving joint proprioception. Evidence for this is lacking in literature.

**Purpose of the Study:**

To compare the myoelectric amplitude of shoulder muscles during push-ups on labile and stable surface.

**Study Design:**

Same subject experimental study.

**Materials and Methods:**

Thirty healthy male subjects in the age group 20-30 years with a mean height of 173.65 cm (± SD 2.56) and a mean weight of 69.9 kg (±SD 0.2) were taken. Surface electromyogram was recorded from triceps, pectoralis major, serratus anterior and upper trapezius while performing push-up and push-up plus exercises, both on labile and stable surface.

**Results:**

Significant increase in muscle activity was observed in pectoralis major and triceps muscle (only during eccentric phase of elbow pushups), while serratus anterior and upper trapezius showed no change in activation level on swiss ball.

**Conclusion:**

The addition of a swiss ball is capable of influencing shoulder muscle activity during push-up variations, although the effect is task and muscle dependent.

## INTRODUCTION

A proper rehabilitation program is essential for the successful treatment of shoulder pathology. The normal gleno-humeral joint demonstrates balance between stability and mobility. The shallow glenoid affords a large degree of motion which is particularly evident in overhead athletes who repeatedly place their arm in positions of extreme ranges of motion; their mobility requires a stable base which is dependent on the strength of muscles around the shoulder joint. These factors must be considered when designing a shoulder rehabilitation program.

Closed chain exercise protocols i.e. pushups and push-up plus are extensively used in rehabilitation of shoulder injuries. Performance on push-ups measures strength and endurance of several upper extremity and trunk muscles.[1] Whether used as an assessment tool or a strengthening exercise, it is important to understand the activation patterns of upper extremity muscles, so that maximal benefits can be obtained. Surface electromyography is used to quantify muscle activity patterns.

An important consideration during shoulder rehabilitation is the addition of surface instability in the form of swiss balls, wobble boards and other labile surfaces. The justification for the use of labile surface is based on its potential for increasing muscular demand required to maintain postural stability, although evidence for this is lacking.[2] It has been demonstrated that individuals will have distinctive movement control behaviors in adapting to stable versus unstable dynamic situations with efferent motor commands resulting in either reciprocal activation or co-contraction patterns of active musculature.[3] There is a small body of evidence to suggest that recruitment patterns of active musculature are affected by the use of an unstable surface provided by the swiss ball. It is also not clear whether performing an exercise on a swiss ball has greater benefit than performing the same exercise on a stable surface. It is often assumed that performing exercises on an unstable surface results in greater muscle activity in an attempt to achieve joint stability. This assumption has a mixed and somewhat sparse support. Garcia *et al.,*[4] showed a consistent increase in selected trunk muscles during curl up on an exercise ball. Similar improvements in joint proprioception have been documented in unstable shoulders following rehabilitation therapy using an unstable surface.[5] Marshall and Murphy[2] showed an increase in the muscle activity when swiss ball was the primary base of support. Lehman *et al.*,[6] have found that replacing an exercise bench for a swiss ball can increase muscle activity, however, the effect is both task and muscle dependent.

Others have shown inconsistent changes with no statistical increase in muscle activity when replacing the swiss ball for an exercise bench during resistance exercises for upper body[7,8] and changes depend upon centre of gravity location relative to unstable surface during bridging[9] or core stability exercises[10]. A study conducted by Lehman[11] showed no statistically significant difference in the mean EMG amplitude when replacing a swiss ball for an exercise bench during push-ups. Thus the influence of unstable surface on the myoelectric activity of shoulder muscles during push-ups and push-up plus position seems to be unclear.

The present study attempts to quantify the effects of unstable and stable surface under the hands during pushups and push-up plus exercises on shoulder muscle activation level. This quantification will help to determine the exact changes in muscle activity and will also help to decide how the muscles responsible for humeral motion can be best exercised in a rehabilitation program.

The muscles examined in the present study are Pectoralis Major [PM], Serratus Anterior [SA], Upper Trapezius [UT] and Triceps [TRI]. The significance of taking these muscles lies in the fact that they are prime movers during a push-up maneuver.

## MATERIALS AND METHODS

Thirty five healthy male subjects with no history of any upper limb or lumbo-sacral problems and without any weight training experience were recruited from a convenience sample of college students out of which five were drop-outs. The exclusion criterion was history of any injury or surgery to upper or lower limb, and female subjects. Their ages ranged between 20-30 years with the mean height of 173.65 cm (± SD 2.56) and the mean weight of 69.9 kg (±SD 0.2). Participants were required to sign an informed consent form prior to study approved by the institution's research board and sanctioned by the university ethical committee. The data collection was undertaken during the period of July-August 2007 under controlled environmental conditions.

To optimize EMG signal collection, participants with a low sub-cutaneous fat from the university population were recruited. The myoelectric activity of triceps, pectoralis major, upper trapezius and serratus anterior were recorded during a series of different variation of push-up exercises.

EMG data was collected using disposable bipolar Ag-Agcl surface electrodes (Trade name KEN NY-1000). NORAXON USA Inc, 1200 EMG unit was used to quantify muscle activity. The EMG signals were amplified by the amplifier system Driver Linx with the input impedance of 10-milli ohm. Gain (fixed) = 1000 Hz, Sampling rate =1000 Hz, Keithley A/D convertor +_ 5V input range, bandwidth=10Hz-500Hz with no notch filter.

**Figure 1:**
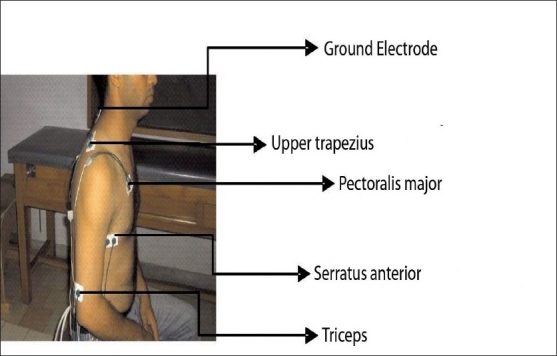
Placement of Electrodes

Before the application of the electrodes skin impedance was reduced by shaving excess body hair if necessary and wiping the skin with ethyl alcohol swabs. All impedance levels were below 5 kohm before data collection started. Pairs of electrode with a diameter of 1cm and center to center spacing of 2.5 cm were applied to the dominant limb: pectoralis major (PM), electrodes were placed four fingerbreadths below clavicle medial to anterior axillary border; triceps (TRI), electrodes were placed at the mid substance of the muscle belly between origin and insertion; upper trapezius (UT) electrode were placed two- third of way between spinous process of seventh cervical vertebrae and acromion; serratus anterior(SA),electrodes were placed parallel to muscles fibers below axilla, anterior to latismus dorsi and posterior to pectoralis major[Figure 1]. All electrodes were placed parallel to the corresponding muscle fibers. A ground electrode was placed over the seventh cervical spinous process.

### Normalization task procedure

Maximum voluntary isometric contractions (MVIC) were performed for each muscle signal before beginning the exercise evaluation.[12] It was done to compare muscle activity across subjects and to give biologically meaningful data. This required the subject to maximally contract each muscle against manual resistance for ten seconds. Three trials of MVIC were taken after adequate familiarization with the procedures in accordance with standard Physical therapy guidelines Daniels and Worthingm.[13] At least 2 min rest was provided between each MVIC contraction.[12]

The MVIC for the PM was performed with the subject supine and shoulder in 60 degrees of abduction, elbow flexed and was asked to horizontally adduct the shoulder, and resistance was given over the wrist against this movement.[13] The MVIC for TRI was performed with elbow and shoulder flexed to 90 degrees and resistance to extension movement was given above the wrist so that an isometric contraction resulted.[13] The maximum UT activation was performed by the seated subject who was instructed to raise his shoulders towards his ears and to hold against maximal resistance given over the shoulders.[13] For SA maximum contraction was performed with arm flexed to 130 degrees of flexion with elbow extended. The subject was instructed to raise his arm forward and resistance to the movement was provided just above the elbow so that an isometric contraction resulted.[13]

### Exercise Protocol

**Figure 2:**
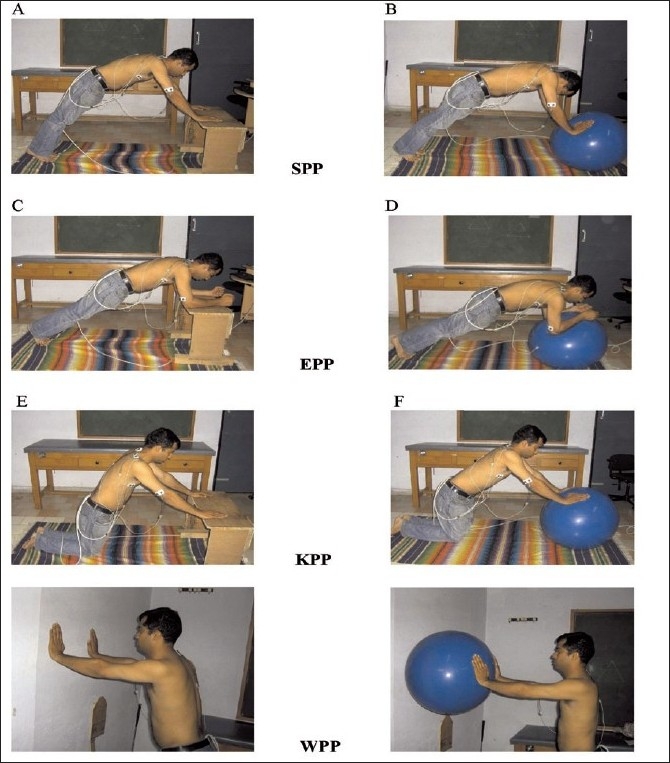
Digital photographs showing concentric phase of different tasks on stable and labile surface

Following the MVIC the participants performed the following exercises in random order (arbitrarily determined by the experimenter), standard push-up [SPP], knee push-up [KPP], elbow push-up [EPP] and wall push-up [WPP] both on labile and stable surface. Modification to SPP were used as they were less challenging and generally advocated earlier in rehabilitation program. The SPP was done with hands shoulder width apart and the arms were allowed to flex at the elbow joints and the body was lowered until the nose touches the floor. The KPP was performed in same way as the SPP except that knees were the distal point of contact with the ground rather than the feet. During EPP elbows were flexed to 90 degrees and upper extremity weight was borne on the elbows. The WPP was performed in a standing position with the hands in contact with the wall [Figure 2]. Positions of the extremities for all exercises were based on creating a resultant shoulder flexion angle of 90 degrees. Subjects were uniformly instructed on performance of each exercise by a single examiner and were allowed to practice a few repetitions until the proper motion and timing was achieved based on visual assessment. Each exercise was completed as a set of three repetitions and a rest period of around three minutes was given between each trial, thus eliminating the potential of fatigue.

### Movement Tasks

The bench height and exercise ball height were standardized and identical to each other. EMG activity of different phases of push-ups and push-up plus both on stable and unstable surface were recorded where by the subjects were made to coordinate their active phases with the beeps set up in EMG appliance, in following manner:

Begin in upright position when EMG collection begins-hold on the position for 3 sec

Eccentric position lasts for 3 sec, hold for 3 sec

Concentric position lasts for 3 sec, hold for 3 sec

Using electrical markings trigger at the start and the end of movement the mean activity for three repetitions was collected.

### EMG Processing

Both MVIC data and myoelectric data from the exercises were processed in the same manner. Using EMG analysis Myoresearch Software Version 2.02, the myoelectrical activity was first demeaned then a root mean square technique was used to smooth the data thus providing a linear envelop of EMG activity. Using the electrical markings the mean activity of the three repetitions was calculated and was then expressed as a percentage of the peak activity found during the maximum voluntary contraction for the corresponding muscle.

Percentage MVIC was calculated as:

$$\mathrm{Percentage MVIC}=\begin{array}{l} \mathrm{Mean amplitude recorded during activity}\\ \mathrm{Maximum voluntary isometric contraction aplitude} \end{array}$$

### Statistical Analysis

Two way analyses of variance (ANOVA) was used to determine the difference between the groups. Post-hoc Tukey test and honestly significant difference [HSD] was performed to find out the reason for significance. The significance level of this study was set at p< 0 .05.

## RESULTS

On comparing the activity levels of muscles on stable and labile surfaces during SPP statistically significant differences were found only for PM [34.72% difference for eccentric and 122% for concentric] with HSD values being 32.66 for eccentric, 106.9 for concentric. Though a difference in the muscle activation was seen in TRI, this did not reach a statistically significant level [Table 1].

**Table 1:** Showing muscle activation and average difference between stable and labile surfaces during standard push-ups

Phases	Surfaces	Muscles	% difference	PM	% difference	UT	% difference	SA	% difference

		triceps							
Eccentric	Stable	67.94±39.46	18.63%	51.16±43.34	34.72	16.46±25.4	0.54	29.55±20.75	15.78
	Labile	86.57±56.37		85.88±71.34		17±14.89		45.33±30.17	
Concentric	Stable	179.4±113.4	86.4%	165.1±117.9	122	93.77±65.3	73.83	45.36±26.95	16.36
	Labile	265.8±113.6		287.1±174.9		167.6±81.94		61.72±40.79	

On comparing the activity levels of muscles during eccentric phase of EPP on stable and labile surfaces, statistically significant differences were found for both TRI[52.82%] and PM [65.19%] based on an HSD value of 40.04 during eccentric phase. While during concentric phases statistically non-significant differences were found [HSD-63] [Table 2].

**Table 2:** Showing muscle activation and average difference between stable and labile surfaces during elbow push-ups

Phases	Surfaces	Muscles	% difference	PM	% difference	UT	% difference	SA	% difference

		triceps							
Eccentric	Stable	45.37±28.71	52.82	50.51±49.66	65.19	11.56±6.9	3.92	58.81±30.13	22.36
	Labile	98.19±66.44		115.7±96.32		15.48±8.2		81.17±53.94	
Concentric	Stable	130.1±101.4	−4.9	149.6±90.45	12.9	27.75±25.24	1.41	48.48±26.93	14.4
	Labile	125.2±100.8		162.5±118.4		29.16±14.67		62.96±23.78	

During KPP on both stable and labile surfaces, non-significant differences were found in the activation level of muscles for both the phases [HSD-20.49 for eccentric, 80.51 for concentric] [Table 3].

**Table 3:** Showing muscle activation and average difference between stable and labile surfaces during knee push-ups

Phases	Surfaces	Muscles	% difference	PM	% difference	UT	% difference	SA	% difference
		**triceps**							
Eccentric	Stable	41.1±29.95	1.16	43.92±25.12	13.84	10.89±10.97	1.64	13.48±8.51	5.26
	Labile	42.26±37.27		57.76±43.83		12.53±16.41		8.74±13.23	
Concentric	Stable	134.7±120.4	78.4	119.2±97.24	58.5	96.95±48.41	55.35	27.43±12.65	7.15
	Labile	213.1±185		177.7±119		152.3±96.96		34.58±16.73	

During WPP on both the surfaces, statistically significant differences in muscle activation was found only for PM during eccentric phase[35.21%] based on an HSD value of 29.73. While during concentric phases, statistically non-significant differences were found [HSD-77.67] [Table 4].

**Table 4:** Showing muscle activation and average difference between stable and labile surfaces during wall push-ups

Phases	Surfaces	Muscles	% difference	PM	% difference	UT	% difference	SA	% difference
		**triceps**							
Eccentric	Stable	60.83±27.08	19.14	51.62±41.69	35.21	41.89±28.06	7.65	33.61±19.43	26.54
	Labile	79.97±47.46		86.83±57.57		49.54±32.4		60.15±35.7	
Concentric	Stable	154.9±103.2	12.9	79.36±68.87	49.34	134.6±73.96	11.5	48.98±24.63	11.36
	Labile	167.8±140		128.7±109		146.1±70.76		60.34±36.81	

## DISCUSSION

In this study, we compared the activation levels of shoulder muscles during the performance of the task on and off swiss ball. The results of this study have demonstrated that the swiss ball can change muscle activity depending on the mechanical nature of the task, i.e. the effect is both task and muscle dependent. Statistically significant differences in the muscle activation level was found only in PM (ecc 34.72%, conc. -122%) during SPP performed on stable and labile surfaces [Table 1]. Previous research[6] demonstrated no change in activation of PM during any push-up variation and increase in TRI muscle activity. The sampling in the current study used healthy male subjects with no weight training experience while the study done by Lehman *et al.*[6] had taken subjects with 6 months of weight training; we believe that this training might have influenced the results. This suggests that the stage of motor learning might influence the activation of key muscles. The results of this study thus suggest that athletes can include both surfaces to vary the degree of muscle activation in PM in the initial phases of training during the performance of SPP.

The UT and SA muscles were not influenced by the addition of the swiss ball during any push-up variations. These results are similar to the findings of Lehman.[11] It may be due to greater redundancy in the motor control of muscles crossing the anterior shoulder. The joint is stabilized by a multitude of muscles and scapular rotation is created by other muscles in addition to above two. Thus merely adding an unstable surface is insufficient to influence all the muscles. Since the center of pressure dispersion of the individual and ball was not measured, the relationship between the amount of instability and the increased muscle activity cannot be evaluated. The mechanical nature of the task i.e. labile surface does appear to be the primary cause of increased activity.[2]

Of note is that performing KPP resulted in no change in muscle activity during both the phases, it may be due to the reason that the knees were the distal point of contact and placed less stability and movement demands on muscles.

Significant increase in the muscle activation level of TRI [52.82%] and PM [65.19%] as shown in Table 2 were found during eccentric phase of EPP. This suggests that the vertical distance from the swiss ball may be an important factor in determining which exercises will see changes in the myoelectric amplitude with the addition of swiss ball.[6] The significant increase in the muscle activation of TRI may be due to the reason that it is a two-joint muscle and has mechanical advantage relative to the length of the forearm as was observed by Lehman *et al.*[6] While the change in PM muscle by the addition of swiss ball may be due to the reason it is a prime mover and is challenged greatest under closed kinematic conditions and thus has difficulty in responding to close kinetic chain conditions on an unstable surface.

The concentric phase and the eccentric phases were also compared, where the up phase of each exercise showed more activity as compared to the down phase. This is in accordance with the EMG force relationship which states that eccentric or lengthening contraction utilizes elastic elements and metabolic processes more efficiently than the concentric contraction. Therefore, for the same amount of muscle tension, an eccentric contraction will require fewer motor units (less overall EMG activity) than a concentric one[14] [Graph 1].

**Figure:**
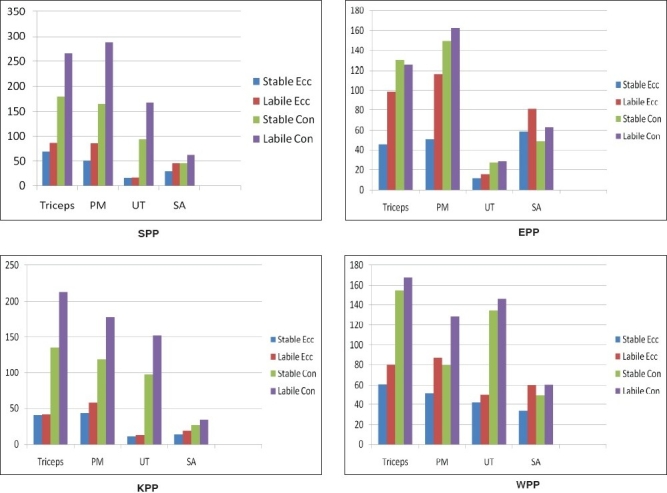
Comparison of Mean %EMG activity of muscles on stable and labile surface for both the phases **Note:** Ecc-Eccentric phase; Conc-Concentric phase

It should also be noted that there is often a range of responses as seen in previous researches. Not every individual responded in the same manner to a change in surface stability. It is possible that there are individual factors that modulate the response to surface stability which also suggests that training may influence the response to instability.[6]

A limitation in explaining our results is the lack of measure of center of pressure dispersion between the individual and the ball; this has been noted by other investigators as well.[2]

## CONCLUSION

Addition of the swiss ball is capable of influencing shoulder muscle activity although the effect is task and muscle dependent. Swiss ball may permit strength training adaptations of the limbs.

### Clinical Relevance

With change of surface, exercise routines can be designed to maximize or minimize muscle activation level depending on the need of patients for clinical training. Swiss balls are often more portable and affordable than a traditional weight bench and may be used to challenge the neuromuscular system and to add variety in the exercise program.
